# Management After Windstorm Affects the Composition of Ectomycorrhizal Symbionts of Regenerating Trees but Not Their Mycorrhizal Networks

**DOI:** 10.3389/fpls.2021.641232

**Published:** 2021-05-14

**Authors:** Petra Veselá, Martina Vašutová, Magda Edwards-Jonášová, Filip Holub, Peter Fleischer, Pavel Cudlín

**Affiliations:** ^1^Department of Carbon Storage in the Landscape, Global Change Research Institute of the Czech Academy of Sciences, Brno, Czechia; ^2^Department of Forest Protection and Wildlife Management, Faculty of Forestry and Wood Technology, Mendel University in Brno, Brno, Czechia; ^3^Department of Carbon Storage in the Landscape, Global Change Research Institute of the Czech Academy of Sciences, České Budějovice, Czechia; ^4^Department of Botany, Faculty of Science, University of South Bohemia, České Budějovice, Czechia; ^5^Department of Integrated Forest and Landscape Protection, Faculty of Forestry, Technical University in Zvolen, Zvolen, Slovakia

**Keywords:** disturbances, diversity, ectomycorrhizal fungi, exploration types, mycorrhizal networks

## Abstract

Due to ongoing climate change, forests are expected to face significant disturbances more frequently than in the past. Appropriate management is intended to facilitate forest regeneration. Because European temperate forests mostly consist of trees associated with ectomycorrhizal (ECM) fungi, understanding their role in these disturbances is important to develop strategies to minimize their consequences and effectively restore forests. Our aim was to determine how traditional (EXT) and nonintervention (NEX) management in originally Norway spruce (*Picea abies*) forests with an admixture of European larch (*Larix decidua*) affect ECM fungal communities and the potential to interconnect different tree species *via* ECM networks 15 years after a windstorm. Ten plots in NEX and 10 plots in EXT with the co-occurrences of Norway spruce, European larch, and silver birch (*Betula pendula*) were selected, and a total of 57 ECM taxa were identified using ITS sequencing from ECM root tips. In both treatments, five ECM species associated with all the studied tree species dominated, with a total abundance of approximately 50% in the examined root samples. Because there were no significant differences between treatments in the number of ECM species associated with different tree species combinations in individual plots, we concluded that the management type did not have a significant effect on networking. However, management significantly affected the compositions of ECM symbionts of Norway spruce and European larch but not those of silver birch. Although this result is explained by the occurrence of seedlings and ECM propagules that were present in the original forest, the consequences are difficult to assess without knowledge of the ecology of different ECM symbionts.

## Introduction

Windstorms represent one of the major factors influencing European temperate forests (Fischer et al., 2013), and their intensity has been increasing during the last few decades (Gregow et al., 2017). Depending on wind severity, the influence of windstorms may extend from single trees to large areas of forests. Large windstorms, causing forest breakdown, affect the forest ecosystem in a complex way. Changes in forest structure lead to overall changes in microhabitat conditions, such as increased solar radiation connected to increased temperatures and changes in water availability (Gömöryová et al., 2008), nutrient cycling (Don et al., 2012), and vegetation cover (Dietz et al., 2020). Because European temperate forests mainly consist of trees living in symbiosis with ectomycorrhizal (ECM) fungi (Martin et al., 2016), these fungal symbionts also face these changes.

Severe windstorms generally lead to decrease in ECM fungal diversity and changes in their community composition (Egli et al., 2002; Ford et al., 2018; Vašutová et al., 2018). Subsequent management practices, such as clear-cut logging, usually enhance these changes as a possible consequence of soil mechanical damage (Ford et al., 2018; Vašutová et al., 2018). The main driver of fungal community shifts is the death of mature trees (Karst et al., 2014; Treu et al., 2014), because ECM fungi are obligate symbionts unable to live without their hosts (Baldrian, 2009; Lofgren et al., 2018). This results in an extinction of ECM fungal species associated with mature trees (= late-successional ECM fungi), while early-successional ECM fungi, with a low demand on photosynthates, survive on seedlings (Egli et al., 2002; Vašutová et al., 2018; Veselá et al., 2019b). Fungal inoculum may further persist in soil for some time as spores or hyphae emanating from dying or recently dead root tips (Simard, 2009; Guignabert et al., 2018). ECM species known to be able to survive or rapidly re-establish after disturbances are, e.g., *Cenococcum* sp., *Laccaria* sp., *Piloderma* sp., *Rhizopogon* sp., *Thelephora* sp., or *Wilcoxina* sp. (Visser, 1995; Jones et al., 2003; Kranabetter et al., 2004; Twieg et al., 2007).

The ability to succeed in young forest stages depends on various functional traits of the ECM fungal species, i.e., on their enzymatic activity, which enables them to decompose various substrates and mobilize nutrients (Velmala et al., 2014; Haas et al., 2018); spore traits which improve their resistance in soil (Jones et al., 2003); and morphology of mycorrhizas represented by exploration type which influences nutrient exploitation from soil and interconnection of trees through the mycorrhizal network (Agerer, 2001; Rosinger et al., 2018). For example, some *Piloderma* species secrete hemicellulases and hydrolytic enzymes involved in litter degradation, while *Wilcoxina* sp. is effective in degrading chitin (Velmala et al., 2014). Higher nitrate uptake and retranslocation to the plant host was also observed in *Laccaria laccata* compared with *Suillus bovinus* (Hobbie et al., 2008). The ascomycete species *Cenococcum geophilum* is supposed to be low carbon demanding due to having a thin mantel and short-distance exploration type (Guignabert et al., 2018; Defrenne et al., 2019), while long-distance explorers belonging to the genus *Rhizopogon* were found to dominate in dry, nutrient-poor areas, since they are able to transport resources from longer distances (Bakker et al., 2006).

Each individual tree can be associated with many ECM fungal species (Bahram et al., 2011), and each ECM fungal species differs in its specificity (Molina and Horton, 2015). ECM fungi with narrow host range (e.g., some *Leccinum*, *Rhizopogon*, and *Suillus* species) are usually restricted to a specific host genus, while ECM fungi with a broad host range (e.g., *Boletus*, *Paxillus*, and *Laccaria* species) are known to be associated with nearly every tree species that form ectomycorrhizae (Molina et al., 1992). ECM fungi with broad host ranges dominate in ECM fungal communities (Molina et al., 1992; Horton and Bruns, 1998; Taudiere et al., 2015; van der Linde et al., 2018; Defrenne et al., 2019; Rog et al., 2020). These fungi can interconnect different tree species and influence nutrient exchange among them (Simard et al., 1997; van der Heiden and Horton, 2009). On the other hand, almost all host tree species harbor in their symbiotic fungal assembly genus-restricted ECM fungi, which provide them exclusive access to resources (Molina et al., 1992). Nevertheless, ECM fungi with a broad host range may also show host preference depending on the ecological context (Lang et al., 2011), while genus-restricted ECM fungi may expand their host range in the absence of their primary host (Lofgren et al., 2018).

The ECM fungal community is a dynamic system interconnecting trees of the same species, but also different tree species through the mycorrhizal network (Simard et al., 1997; Klein et al., 2016; Rog et al., 2020). This phenomenon is not negligible when considering that up to approximately 40% of fine-root carbon can originate from mycorrhizal network exchange (Klein et al., 2016). Not only carbon can be transferred through the mycorrhizal network, but also water and other nutrients, or signaling molecules (Gorzelak et al., 2015). The mycorrhizal network is influenced by tree physiology, e.g., photosynthetic and growth rates, nutrient content, forest age, or infestation (Simard et al., 1997, 2012; van der Heiden and Horton, 2009; Song et al., 2015; Simard, 2018); the phylogenetic relationships among trees involved in the network (Rog et al., 2020); environmental conditions (Rosinger et al., 2018; Defrenne et al., 2019); and also by the ECM fungi themselves which protect their carbon donors (Song et al., 2015). This system is expected to be very dynamic and vulnerable to large disturbances, as demonstrated by Pec et al. (2020).

Severe windstorm damaged approximately 12,000 ha of mountain spruce forest in the Tatra Mts. (Slovakia) in 2004 (Fleischer, 2008). In most of the affected area, the fallen trees were extracted (EXT), while a small part was left for natural succession (NEX). To bring new perspectives to the discussion on the suitability of subsequent management, we were interested in how the windstorm and following timber harvest affected the ECM fungal community and its potential to form mycorrhizal networks among regenerating tree species, Norway spruce, European larch, and silver birch. We hypothesized that ECM fungi with a broad host range will prevail in plots with traditional management (e.g., fallen tree extraction) and that different tree species will therefore share a high percentage of ECM fungal species. On the other hand, plots left for natural succession will harbor more ECM fungi with narrow host range associated with target tree species, with fewer ECM fungal species shared among them. We also hypothesized that the number of ECM fungal species would decrease in managed plots due to soil compaction and mechanical damage to seedlings that survive and the ECM fungal extramatrical mycelium.

## Materials and Methods

### Study Site

The research plots are situated in the Tatra Mts. (Slovakia) in the Tatra National Park (Supplementary Figure 1). This area was affected by severe windstorm in 2004 during which about 12,000 ha of forest was destroyed. The area was originally covered by a Norway spruce (*Picea abies*) forest with an admixture of European larch (*Larix decidua*), and Scotch pine (*Pinus sylvestris*). Currently, this area is dominated by young trees of Norway spruce, birches (*Betula* sp.), European larch, and European rowan (*Sorbus aucuparia*) in variable proportions depending on the microhabitat conditions. Locally, Scots pine, goat willow (*Salix caprea*), and silver fir (*Abies alba*) are present. The area is characterized by a cold climate and has a mean annual temperature of 5.3°C and a mean annual precipitation of 833 mm (Fleischer, 2008). The soils in the plots are dystric cambisols on glacial moraine deposits with a loamy sand texture, an acidic pH of approximately 3.0 (Don et al., 2012), and carbon and nitrogen percentages of approximately 11.9 and 0.6%, respectively (Vašutová et al., 2018).

Following this disturbance event, permanent research sites with different management regimes were established in 2005, and each research site is about 100 ha wide (Fleischer, 2008). Within the current research, we studied two treatments: (i) site with traditional management (EXT), where fallen trees were extracted and the locality was consequently reforested, and (ii) a site without management (NEX) left for natural succession, in which fallen trees were left. Ten circular plots (*d* = 12.6 m and *P* = 125 m^2^) per treatment were selected so that there were at least two individuals of each tree species of interest, Norway spruce, European larch, and silver birch (*Betula pendula*), and the presence of other ECM tree species was avoided to minimize influencing the ECM fungal community.

### Ectomycorrhizae on Tree Roots

ECM root samples (each consists of about 100 root tips) from 20 plots were collected during 2 years: three plots per treatment in September 2018 and seven plots per treatment in September 2019 (Supplementary Table 1). In each plot, two individuals for each tree species were selected, and three root samples from each individual tree were collected. The cover of trees, shrubs, herbs, mosses, and dead wood was visually estimated (Supplementary Table 1). Dead wood was classified into five categories (lying logs, piles of branches, root collars, stubs, and stumps).

The height and perimeter of each selected tree were measured (Supplementary Table 1). For each root sample, its depth, soil horizon, distance from the tree stem, and direction to other tree species were recorded (Supplementary Table 1). In total, 60 root samples from each tree species per treatment (2 trees × 3 root samples per plot × 10 plots) were sampled, with the exception of Norway spruce (due to the misidentification of one of the root samples, only 59 samples were analyzed).

The identity of the roots was guaranteed by direct tracing from a tree stem; in disputable cases, the identity was confirmed using chloroplast DNA sequence comparison against the GenBank database (Benson et al., 2007) using MegaBlast algorithm, with 97% similarity threshold as a molecular species criterion.

### Processing of Root Samples

Each root sample was carefully washed under tap water, and all attached soil particles were removed using tweezers in a Petri dish with water. The root samples were examined under a stereomicroscope and documented (Canon 1,000D, Tokyo, Japan). The ECM root tips of each root sample were sorted into morphotypes according to their morphological features: color, presence of emanating hyphae and rhizomorphs, cystidia, and ramification (Agerer, 1991; Agerer and Rambold, 2004–2015). Each morphotype was excised and stored in a tube with silica gel. At least one morphotype per tree species and treatment was then used for molecular identification.

### DNA Extraction and Molecular Identification

Genomic DNA was extracted from the selected ECM root tips using the DNeasy Plant Mini Kit (Qiagen, Hilden, Germany) according to the manufacturer’s instructions. The extracted DNA was then used as a template for PCR amplification of the rDNA ITS region with the primer pairs ITS1F/ITS4 (White et al., 1990; Gardes and Bruns, 1993) for fungal species identification and of the noncoding region of chloroplast DNA with the primers *trn*L/*trn*F (Taberlet et al., 1991) for plant species identification. In case that we obtained multiple fungal amplicons from a root tip, we used primer pairs specific for basidiomycetous (ITS1F/ITS4B; Gardes and Bruns, 1993; Nikolcheva and Bärlocher, 2004) or ascomycetous fungi (ITS1/ITS4A; White et al., 1990; Nikolcheva and Bärlocher, 2004). When the amplicon separation was unsuccessful, we cut the bands directly from gel using QIAquick Gel Extraction Kit (Qiagen). PCR was performed in a 25-μl reaction mixture containing 1× reaction buffer (Bioline, London, United Kingdom), 500 nM of each primer, 0.75 U of MyTaq DNA polymerase (Bioline), and ∼20 ng of template DNA filled with dH_2_O up to 25 μl. The amplification conditions were 94°C for 2.5 min, followed by 37 cycles at 94°C for 30 s, 55°C for 40 s, 72°C for 30 s, and a final extension at 72°C for 4.5 min for the fungal amplicons; 94°C for 2 min, followed by 35 cycles at 94°C for 30 s, 56°C for 30 s, 72°C for 1.2 min, and a final extension at 72°C for 10 min for the plant amplicons. Sequencing was performed by Macrogen Inc., Amsterdam, Netherlands, on an ABI 3730 XL automated sequencer (Applied Biosystems, Foster City, CA, United States).

### Molecular Data Analysis

Sequence chromatograms were analyzed using Chromas Lite v.2.6.5 (Technelysium Pty Ltd, Brisbane, Australia), and the sequences were blasted against the UNITE (Abarenkov et al., 2010) and GenBank (Benson et al., 2007) public databases. A 97% similarity threshold was used as a molecular species criterion and 90% similarity threshold as a molecular genus criterion (Nilsson et al., 2019; Vu et al., 2019). The sequences were deposited in GenBank (accession numbers: MT908274–MT908330). Based on the molecularly identified morphotypes, species names were given to all the documented morphotypes.

The assignment of ECM taxa into exploration types was made according to Agerer (2001), Agerer and Rambold (2004–2015), and our observations (Supplementary Table 2).

### Statistical Analysis

The numbers of ECM fungal species shared by all tree species, a combination of two tree species, or unique for a single tree species were calculated for the entire dataset, EXT and NEX treatments and separately for each plot. The same approach was used in assessing the number of species with the exploration types with rhizomorphs (long-distance exploration type, medium-distance fringe exploration type, and medium-distance smooth exploration type), which could in particular provide networking.

The tree heights and perimeters per tree/tree species for the NEX and EXT treatments and the numbers of ECM fungal species per tree/tree species for the NEX and EXT treatments were tested by analysis of variance with Tukey’s *post hoc* contrasts in R 3.6.2 (R Development Core Team, 2019).

Rarefaction curves, using R package mobr (McGlinn et al., 2018), were prepared for all tree species within the NEX and EXT treatments (Supplementary Figure 2).

Three datasets were prepared for relating the plot variables (*n* = 20), trees (*n* = 120), and root samples (*n* = 359). Presence/absence data (roots) and number of root samples with each morphotype (trees, plots) were used to avoid difficulties in the proper identification of not well-developed morphotypes. The datasets were separately analyzed by multivariate methods in Canoco 5 program (ter Braak and Šmilauer, 2012), using detrended correspondence analysis (DCA) and canonical correspondence analysis (CCA). DCA was used to compare the ECM fungal species compositions in plots, trees, and roots. The year of sampling was used as a covariate. CCA was used to identify (i) the effects of management, vegetation cover, and geographical position on ECM fungal species composition in plots; (ii) the effects of management, tree species, height, and perimeter on ECM fungal species composition in trees; and (iii) the effects of management, tree species, distance from tree stem, and soil horizon on the ECM fungal species composition in roots. The dataset trees was divided based on tree species identity, and the resulting datasets were further analyzed separately. Various environmental variables were tested as explanatory variables in CCA analyses. Year (for all datasets) and plot identifier in interaction with year (only for datasets roots and trees) were used as covariates. We used hierarchical split-plot design for permutations and permutated whole plots (i.e., keeping the data from one plot together) for testing the effect of management. These whole plots were permuted at random within the covariate year, and the two split plots (trees) were not permuted. The effect of tree species was tested by split-plot permutations (within the whole plot, i.e., the data from different tree species were permutated within the plot). The effects of explanatory variables were summarized in partial analyses after removing the effects of covariates. The Monte Carlo permutation test was used and only significant variables were selected.

The effects of tree species and management on the number of ECM fungal species for tree were analyzed by linear mixed-effects model fit by REML using the lme function in nlme package in R 3.6.2., with plot as a random effect. The effect of plot was tested by comparing the model without plot (made by gls function) and the model with plot (made by lme function) by function ANOVA.

The numbers of shared and specific ECM fungal species in NEX and EXT treatments on *P. abies*, *L. decidua*, and *B. pendula* were presented by Venn diagrams made in web application BioVenn (Hulsen et al., 2008).

Two root samples (19Pa_2b and 2Ld_1a) and one tree sample (9Pa) were excluded from analysis as outliers.

## Results

### ECM Fungal Species and Potential ECM Networks

In total, 57 ECM taxa were detected (Supplementary Table 2): 51 taxa were assigned at the species level, two at the genus level, and four at the order level. There were no significant differences in the number of ECM fungal species per tree between the NEX and EXT treatments (*p* > 0.05, Table 1). However, differences among tree species were detected; silver birch had significantly higher number of ECM fungal species per tree than Norway spruce and European larch (*p* < 0.005), and Norway spruce and European larch did not differ significantly (*p* > 0.05, Table 1).

**TABLE 1:** Mean number of ectomycorrhizal (ECM) fungal species per tree, mean tree height, and mean tree perimeter in NEX (plots without management) and EXT (plots with traditional management) treatments and in Norway spruce, European larch, and silver birch.

	**Number of ECM fungal species/tree**	**Mean tree height (m)**	**Mean tree perimeter (cm)**
NEX	4.5 ± 0.3	3.7 ± 0.2	5.8 ± 0.5
EXT	4.4 ± 0.2	4.8 ± 0.3	7.9 ± 0.6
		NEX/EXT	NEX/EXT
Norway spruce	4.3 ± 1.4	3.0 ± 0.2/3.9 ± 0.4	4.3 ± 0.4/5.6 ± 0.7
European larch	3.2 ± 2	3.1 ± 0.3/4.7 ± 0.4	4.3 ± 0.6/7.1 ± 0.7
silver birch	5.5 ± 1.4	5.1 ± 0.5/5.9 ± 0.4	8.8 ± 1.1/11.1 ± 1.1

The tree heights and perimeters within a treatment were significantly greater for silver birch than for the other tree species (*p* < 0.001, Table 1). In EXT, tree heights (*p* < 0.001, Table 1) and perimeters (*p* < 0.005, Table 1) were significantly greater than in NEX.

**FIGURE 1:**
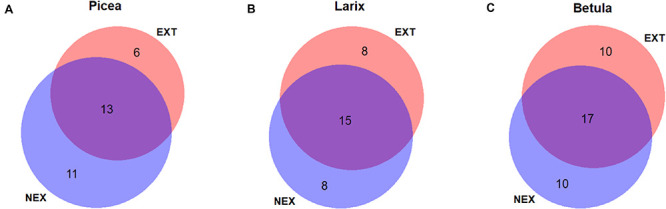
Venn diagram displaying the numbers of shared and specific ectomycorrhizal (ECM) fungal species in NEX (plots without management) and EXT (plots with traditional management) treatments on **(A)** *Picea abies*, **(B)** *Larix decidua*, and **(C)** *Betula pendula*.

Fourteen ECM taxa were found in all tree species, five species were detected in Norway spruce and European larch, seven in Norway spruce and silver birch, five in European larch and silver birch, and eight were unique to Norway spruce, 10 to European larch, and 14 to silver birch. When comparing the numbers of ECM fungal species in single tree species between the NEX and EXT treatments (Figure 1), only Norway spruce had a lower number of ECM fungal species in EXT (six species) compared with NEX (11 species). When focusing on the potential mycorrhizal networking among trees in individual plots, we found no significant differences between NEX and EXT in the numbers of ECM fungal species that were shared by all host trees, between individual pairs of host trees, or associated with a single host tree only.

Overall, 12 ECM species belonged to contact exploration type, 15 species to short-distance type, eight species to medium-distance smooth type, 12 species to medium-distance fringe type, and 11 species to long-distance type (Supplementary Table 2). When considering only the numbers of rhizomorph-forming ECM fungal species, i.e., medium-distance and long-distance exploration types, more ECM species shared by all tree species were found in NEX compared with EXT, but these differences were not significant (Supplementary Table 3). In case of NEX treatment, the potential networkers were *Cortinarius bataillei*, *C. croceus*, *Paxillus involutus*, *Piloderma olivaceum*, and *Thelephora terrestris*, and in case of EXT, the potential networkers were *P. olivaceum* and *T. terrestris*.

There were also no significant differences in the numbers of shared species among combinations of two tree species or in single tree species between NEX and EXT treatments.

Five ECM fungal species dominated in both treatments: *Cenococcum geophilum*, *Lactarius rufus*, *P. involutus*, *P. olivaceum*, and *T. terrestris* (Table 2). Most of these species were detected in all tree species and together accounted for 50.1% in the NEX plots and 51.7% in the EXT plots. The potential of these species to form mycorrhizal networks differed only for the case of *P. involutus*, which was absent in EXT on Norway spruce.

**TABLE 2:** Dominant ECM fungal species present in NEX (plots without management) and EXT (plots with traditional management) treatments.

**Dominant ECM fungi (% of the root samples)**	**Exploration type**	**NEX**	**EXT**
*Cenococcum geophilum*	SD	11.8	13.6
*Lactarius rufus*	C	8.6	15.7
*Paxillus involutus*	LD	12.1	9.3
*Piloderma olivaceum*	MDf	8.3	6.7
*Thelephora terrestris*	MDs	9.3	6.4

*C, contact type; LD, long-distance type; MDf, medium-distance fringe type; MDs, medium-distance smooth type; SD, short-distance type.*

ECM fungal species that were unique to individual tree species were generally rare. For Norway spruce, *Piloderma bicolor* (0.5%, both NEX and EXT) was present in both treatments; in NEX, *Amphinema byssoides* (1.8%), Helotiales1 (0.8%), and *Imleria badia* (0.5%) were present; in EXT, *Tylopilus felleus* (0.8%) was present. For European larch, *Suillus gravillei* (in NEX 0.8% and in EXT 2.6%) was present in both treatments; in NEX, *Cortinarius cinnamomeus* (0.8%) was present; in EXT, *C. parvanulatus* (0.5%), *S. cavipes* (0.3%), and *S. viscidus* (1.8%) were present. For silver birch, *Leccinum holopus* (1.5% in both NEX and EXT), *L. variicolor* (in NEX 3.5% and in EXT 3.3%), and *Meliniomyces bicolor* (in NEX 0.5% and in EXT 0.8%) were present in both treatments; in NEX, *C. porphyropus* (0.5%), *Inocybe soluta* (0.8%), *Laccaria proxima* (0.5%), and *L. glyciosmus* (2.3%) were present; in EXT, *Amanita muscaria* (0.5%) and *Clavulina* sp. (0.5%) were present.

### Management Effect on ECM Fungal Species Composition

Management significantly influenced the ECM fungal species composition in the studied plots and explained 11.7% of the detected variability (Table 3). The other studied environmental variables (vegetation and dead wood cover, geographic position, morphometric parameters of host trees—height, perimeter, and root placement in soil) had no significant effect.

**TABLE 3:** Results of canonical correspondence analyses (CCA) of ECM fungal community.

**Dataset**	**Explanatory variables**	**Covariates**	**Explained variability**	**Pseudo-*F***	***p***
Roots	Tree species	Plot × year	4.7	7.9	0.001
	Management type	Year	1.0	3.4	0.001
	Soil horizon	Plot × year	2.1	2.2	0.001
Trees	Tree species	Plot × year	9.9	5.4	0.001
	Management type	Year	2.2	2.4	0.003
*Picea abies*	Management type	Year	5.3	2.1	0.003
*Larix decidua*	Management type	Year	4.6	1.8	0.019
*Betula pendula*	Management type	Year	*3.6*	*1.4*	*0.193*
Plots	Management type	Year	11.7	2.2	0.001

*Datasets were based on the presence of ECM fungal species in plots, trees, and roots. Variations in ECM fungal community data explained by selected explanatory variables were summarized after removing the effects of covariates (year, interaction of plot and year).*

**FIGURE 2:**
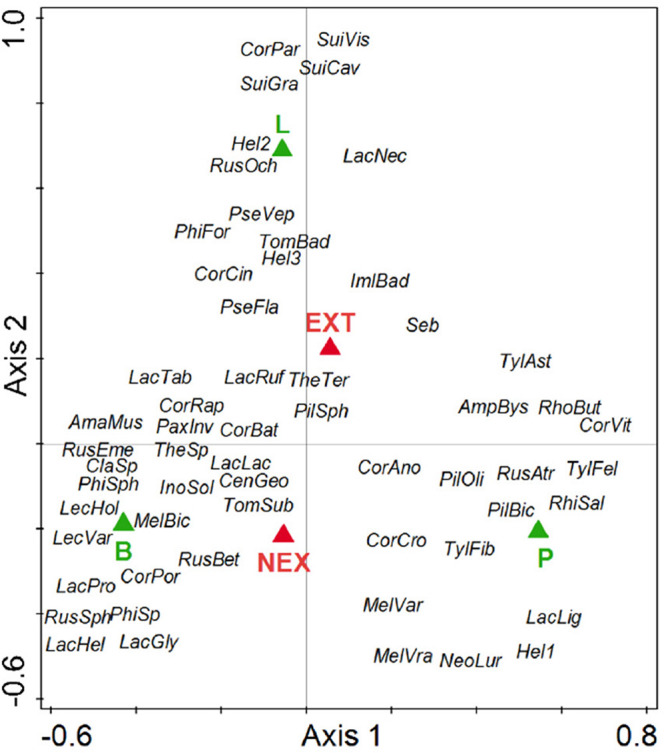
Ordination diagram from canonical correspondence analysis (CCA) of ECM fungal species presence on tree species (P—*Picea abies*, L—*Larix decidua*, and B—*Betula pendula*) in NEX (plots without management) and EXT (plots with traditional management) treatments. Species: AmaMus—*Amanita muscaria*, AmpBys—*Amphinema byssoides*, CenGeo—*Cenococcum geophilum*, ClaSp—*Clavulina* sp., CorAno—*Cortinarius anomalu*, CorBat—*Cortinarius bataillei*, CorCin—*Cortinarius cinnamomeus*, CorCro—*Cortinarius croceus*, CorPar—*Cortinarius parvannulatus*, CorPor—*Cortinarius porphyropus*, CorRap—*Cortinarius raphanoides*, CorVit—*Cortinarius vitiosus*, Hel1—Helotiales1, Hel2—Helotiales2, Hel3—Helotiales3, ImlBad—*Imleria badia*, InoSol—*Inocybe soluta*, LacLac—*Laccaria laccata*, LacPro—*Laccaria proxima*, LacGly—*Lactarius glyciosmus*, LacHel—*Lactarius helvus*, LacLig—*Lactarius lignyotus*, LacNec—*Lactarius necator*, LacRuf—*Lactarius rufus*, LacTab—*Lactarius tabidus*, LecHol—*Leccinum holopus*, LecVar—*Leccinum variicolor*, MelBic—*Meliniomyces bicolor*, MelVar—*Meliniomyces variabilis*, MelVra—*Meliniomyces vraolstadiae*, NeoLur—*Neoboletus luridiformis*, PaxInv—*Paxillus involutus*, PhiFor—*Phialocephala fortinii*, PhiSph—*Phialocephala sphaeroides*, PilBic—*Piloderma bicolor*, PilOli—*Piloderma olivaceum*, PilSph—*Piloderma sphaerosporum*, PseFla—*Pseudotomentella flavovirens*, PseVep—*Pseudotomentella vepallidospora*, RhiSal—*Rhizopogon salebrosus*, RhoBut—*Rhodocollybia butyracea*, RusAtr—*Russula atrorubens*, RusBet—*Russula betularum*, RusEme—*Russula emetica*, RusOch—*Russula ochroleuca*, RusSph—*Russula sphagnophila*, Seb—Sebacinales, SuiCav—*Suillus cavipes*, SuiGra—*Suillus grevillei*, SuiVis—*Suillus viscidus*, TheSp—*Thelephora* sp., TheTer—*Thelephora terrestris*, TomBad—*Tomentella badia*, TomSub—*Tomentella sublilacina*, TylFel—*Tylopilus felleus*, TylAst—*Tylospora asterophora*, and TylFib—*Tylospora fibrillosa*.

**FIGURE 3:**
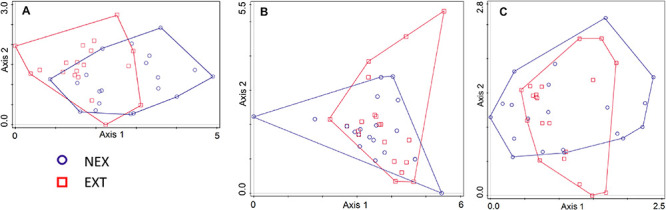
Ordination diagram from detrended correspondence analysis (DCA) with the ECM fungal species composition in NEX (plots without management) and EXT (plots with traditional management) treatments for single tree species: **(A)** *Picea abies*, **(B)** *Larix decidua*, and **(C)** *Betula pendula*.

**FIGURE 4:**
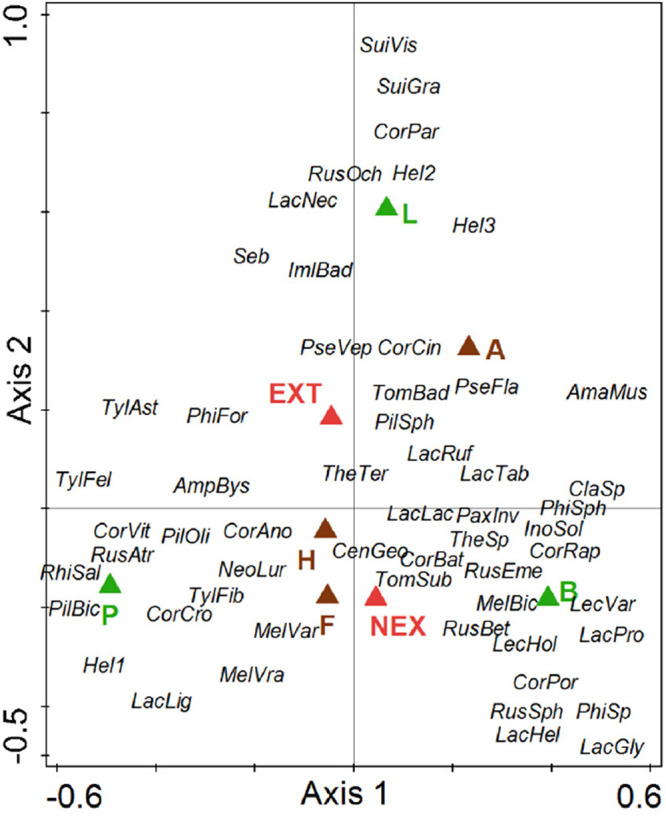
Ordination diagram from canonical correspondence analysis (CCA) of ECM fungal species presence on tree roots in NEX (plots without management) and EXT (plots with traditional management) treatments for single tree species (P—*Picea abies*, L—*Larix decidua*, and B—*Betula pendula*) differentiated according to organic (F, H) and organo-mineral (A) soil horizons. Species: AmaMus—*Amanita muscaria*, AmpBys—*Amphinema byssoides*, CenGeo—*Cenococcum geophilum*, ClaSp—*Clavulina* sp., CorAno—*Cortinarius anomalu*, CorBat—*Cortinarius bataillei*, CorCin—*Cortinarius cinnamomeus*, CorCro—*Cortinarius croceus*, CorPar—*Cortinarius parvannulatus*, CorPor—*Cortinarius porphyropus*, CorRap—*Cortinarius raphanoides*, CorVit—*Cortinarius vitiosus*, Hel1—Helotiales1, Hel2—Helotiales2, Hel3—Helotiales3, ImlBad—*Imleria badia*, InoSol—*Inocybe soluta*, LacLac—*Laccaria laccata*, LacPro—*Laccaria proxima*, LacGly—*Lactarius glyciosmus*, LacHel—*Lactarius helvus*, LacLig—*Lactarius lignyotus*, LacNec—*Lactarius necator*, LacRuf—*Lactarius rufus*, LacTab—*Lactarius tabidus*, LecHol—*Leccinum holopus*, LecVar—*Leccinum variicolor*, MelBic—*Meliniomyces bicolor*, MelVar—*Meliniomyces variabilis*, MelVra—*Meliniomyces vraolstadiae*, NeoLur—*Neoboletus luridiformis*, PaxInv—*Paxillus involutus*, PhiFor—*Phialocephala fortinii*, PhiSph—*Phialocephala sphaeroides*, PilBic—*Piloderma bicolor*, PilOli—*Piloderma olivaceum*, PilSph—*Piloderma sphaerosporum*, PseFla—*Pseudotomentella flavovirens*, PseVep—*Pseudotomentella vepallidospora*, RhiSal—*Rhizopogon salebrosus*, RhoBut—*Rhodocollybia butyracea*, RusAtr—*Russula atrorubens*, RusBet—*Russula betularum*, RusEme—*Russula emetica*, RusOch—*Russula ochroleuca*, RusSph—*Russula sphagnophila*, Seb—Sebacinales, SuiCav—*Suillus cavipes*, SuiGra—*Suillus grevillei*, SuiVis—*Suillus viscidus*, TheSp—*Thelephora* sp., TheTer—*Thelephora terrestris*, TomBad—*Tomentella badia*, TomSub—*Tomentella sublilacina*, TylFel—*Tylopilus felleus*, TylAst—*Tylospora asterophora*, and TylFib—*Tylospora fibrillosa*.

Regarding the community composition of ECM fungal species based on the tree dataset, the only significant variables were tree species and management type (Table 3 and Figure 2). The morphometric parameters of trees (height and perimeter) had no effect on the ECM fungal species composition. When the tree species were analyzed separately, only the ECM fungal species compositions of Norway spruce and European larch were affected by the management type (Table 3 and Figures 3A,B). In Norway spruce trees, *C. croceus*, *Meliniomyces vraolstadiae*, *P. involutus*, *T. terrestris*, and *T. fibrillosa* were more abundant in NEX, whereas *A. byssoides*, *L. rufus*, and *T. asterophora* were more abundant in EXT. In European larch trees, *C. bataillei*, *L. laccata*, *P. olivaceum*, and *T. terrestris* were more abundant in NEX and *Suillus* spp. in EXT. The ECM fungal species composition of silver birch was not influenced by the studied variables (Table 3 and Figure 3C). The ECM fungal species composition, based on the root sample dataset, was significantly affected by tree species, management type, and soil horizon (Table 3 and Figure 4).

## Discussion

### Potential Mycorrhizal Networks and ECM Fungal Diversity

Our study on the effect of different management practices on ECM fungal communities of regenerating trees in the post-windstorm area in the Tatra Mts. revealed no effect on dominant ECM fungi and their potential to form mycorrhizal networks. For both treatment types, five ECM fungal species with broad host range dominated and included both early-colonizers (*C. geophilum*, *P. involutus*, and *T. terrestris*) and late-successional ECM fungi (*L. rufus* and *P. olivaceum*; Visser, 1995; Twieg et al., 2007; Kim et al., 2021). The windstorm apparently affected forest ecosystem in such an extreme manner that the management type produced only insignificant adjustments and had only limited influence on the potential to form ECM networks.

In contrast to our hypothesis, we also found almost no difference in the total number of potentially shared ECM fungal species among different tree species between the traditionally managed plots (EXT) and plots without management (NEX). Even if we focus on ECM species with exploration types with rhizomorphs, we found no significant differences, although the numbers of species shared by all tree species slightly differ in favor of NEX. Exploration types with rhizomorphs are more carbon demanding compared with contact and short-distance types (Defrenne et al., 2019; Wasyliw and Karst, 2020); on the other hand, they are able to form common mycelial networks and exploit water and nutrients from a larger area (Simard et al., 2003; Bakker et al., 2006). So, a higher number of these species per plot in NEX could be explained by lower damage to the mycelium caused by management practices, since hyphae are able to emanate from dying or recently dead root tips for a short time (Simard, 2009).

Norway spruce was the only tree species, which showed a higher number of specific ECM fungal species in NEX than in EXT (Figure 1). This difference is also evident from rarefaction curves (Supplementary Figure 2). In contrast to other tree species in our study, Norway spruce is a shade-tolerant species, which is able to regenerate under mature forest canopies (Metslaid et al., 2007). Moreover, Norway spruce requires an adequate water supply. Gömöryová et al. (2008) detected lower soil moisture levels 2 years after the windstorm in EXT compared with NEX, which could, along with the increased insolation following tree removal, negatively affect spruce fitness. The better Norway spruce seedling fitness in NEX (although not supported by morphological characteristics) combined with the connectivity of the original forest could explain the differences observed. In contrast, both silver birch and European larch possessed the same numbers of specific ECM symbionts in both treatments. The highest ECM fungal species richness in silver birch can be the consequence of its larger dimensions related to its pioneer strategy and higher adaptability to clear-cut sites (Dubois et al., 2020). Similar findings were also reported by Twieg et al. (2007), who detected more ECM fungal species on paper birch than Douglas fir in young stands after clear-cut logging. However, in that case, the higher species richness of paper birch was also attributed to the presence of birch stumps.

No or small differences in quantitative parameters between treatments could be also related to a common problem in evaluating the responses of fungal communities to environmental factors or management. Generally, most fungal communities are composed of a small number of dominant species with a wide ecological valence that only respond to very pronounced gradients. These species are accompanied by few subdominant species and numerous rare species. Due to their rarity and the patchiness of ECM fungi dropping after 3–4 m (Lilleskov et al., 2004; Pickles et al., 2012), it is impossible to disentangle the random occurrence of these species and their response to environmental factors without including many more research plots and more intensive root sampling. Rosinger et al. (2018) found the majority of ECM taxa in one or two plots out of approximately 100 plots. While the overall fungal diversity in temperate forests is high, the species diversity at small scales is low and does not usually exceed 10–20 species (Tedersoo et al., 2003; Anderson et al., 2014). Thus, the theoretical absence of a few species for a total area of 10 times 125 m^2^ could indicate larger losses at larger scales. However, this hypothesis must be verified by further research focusing on the ecology of rare ECM fungal species, in which all the above mentioned limitations would be taken into consideration (i.e., rarity and patchiness of ECM fungal species and sampling effort). The combination of fruit body monitoring together with environmental soil sequencing could be a promising nondestructive method, but unfortunately without data on the associations with host trees.

### Effect of Management on ECM Fungal Species Composition 15 Years After a Windstorm

Interestingly, management is the only significant variable that explains ECM fungal species composition 15 years after a windstorm on the studied plots and for Norway spruce and European larch trees. The similarity of the ECM fungal communities did not reflect the phylogenetic relationships of the studied trees, which contradicts to the observations of Rog et al. (2020).

The observed differences in ECM fungi composition between NEX and EXT in Norway spruce are difficult to explain because there are no clear patterns in exploration types (Agerer, 2001) or spore characteristics (Bernicchia and Gorjón, 2010; Knudsen and Vesterholt, 2012). We also do not expect differences in ability to spread, because both treatments contain species with pileate and resupinate fruit bodies (Lilleskov and Bruns, 2005; Halbwachs et al., 2016). However, the affinity of *T. fibrillosa* and *T. terrestris* for NEX and of *A. byssoides* and *T. asterophora* for EXT has been previously observed in 4–9-year-old seedlings from a similar area in the Tatra Mts. (Vašutová et al., 2018), and these differences persist in Norway spruce between treatments. These differences are difficult to evaluate without detailed knowledge of the ECM fungal ecology and physiology. European larch trees are often occupied by *C. bataillei*, *L. laccata*, *P. olivaceum*, and *T. terrestris* in NEX and by *Suillus* spp. in EXT. Coleman et al. (1989) found several *Suillus* spp. to be drought tolerant, and in contrast, *L. laccata* was not drought tolerant, which could partly explain the observed differences.

Although the studied sites were chosen to be the same as each other and the lack of differences in silver birch-associated ECM fungal community supported a good experimental design, we could not fully exclude the differences in ECM fungal community of original Norway spruce forest with European larch and Scotch pine admixtures (*Lariceto*–*Picetum*) that persisted in spores and as mycorrhizae on surviving seedlings. It would be interesting to determine whether these differences will persist 10–15 years later when ECM fungal diversity should be saturated after canopy closure (Visser, 1995; Twieg et al., 2007). More replications under different local conditions and with other tree species are necessary to generalize our results.

Regarding the effects of vertical distribution on the ECM fungal species composition, *Tylospora* spp., *Amphinema* spp., and *Meliniomyces* spp. showed an affinity for an organic layer, and *Tomentella* and *Pseudotomentella* spp. showed an affinity for an organo-mineral layer (Figure 4), as was previously observed by Tedersoo et al. (2003). They detected athelioid and thelephoroid species as indicators of organic layers, but *Tomentella* spp. were present in all horizons depending on the species. These findings support the importance of vertical niche partitioning, as was already discussed by Bahram et al. (2015).

In conclusion, different post-windstorm management practices do not significantly influence the potential for mycorrhizal networking among Norway spruce, European larch, and silver birch, which is mostly provided by common ECM fungal species with broad ecological niches. Management type affects the ECM fungal species compositions on Norway spruce and European larch; however, the consequences are difficult to assess without knowledge of the ecology and physiology of the affected ECM fungi. Norway spruce in NEX harbored a higher number of unique species than in EXT, but extremely intensive sampling is required to evaluate the possible loss of rare species due to traditional management.

## Data Availability Statement

The datasets presented in this study can be found in online repositories. The names of the repository/repositories and accession number(s) can be found in the article/Supplementary Material.

## Author Contributions

PV, MV, FH, PC, and PF designed the research. PV, MV, and FH performed the research and collected the data. ME-J analyzed the data. PV, MV, and ME-J interpreted the data. PV and MV wrote the manuscript with the assistance from all co-authors. All authors contributed to the article and approved the submitted version.

## Conflict of Interest

The authors declare that the research was conducted in the absence of any commercial or financial relationships that could be construed as a potential conflict of interest.

## Supplementary Material

Supplementary Figure 1 Map of the research area, Tatra Mountains National Park (TANAP), Slovakia (SK). Two management treatments, site without management (NEX) and site with traditional management (EXT), each with ten research plots.

Supplementary Figure 2 Rarefaction curves showing the ECM species richness on tree species (P – *Picea abies*, L – *Larix decidua*, and B – *Betula pendula*) in NEX (plots without management) and EXT (plots with traditional management) treatments.

Supplementary Table 1 Characteristics of the plots in NEX (plots without management) and EXT (plots with traditional management) treatments (geographic position, cover of trees, cover of dominant herbs and shrubs, and dead wood cover); characteristics of the selected trees (height and perimeter); and characteristics of selected roots (depth, soil horizon, distance from the tree stem, and direction to other tree species).

Supplementary Table 2 List of ECM taxa, including exploration of each taxa, detected on root samples of the studied tree species (*Picea abies*, *Larix decidua*, and *Betula pendula*), including the numbers of root samples with taxon presence divided into NEX (plots without management) and EXT (plots with traditional management) treatments. For each taxon, the GenBank Accession Number, closest hit and percentage of similarity are shown.

Supplementary Table 3 Presence/absence matrix showing rhizomorph-forming ECM fungal species in the plots in NEX (plots without management) and EXT (plots with traditional management) treatments.
