# Supplementary material for: Management After Windstorm Affects the Composition of Ectomycorrhizal Symbionts of Regenerating Trees but Not Their Mycorrhizal Networks
Source: Front Plant Sci. 2021 May 14;12:641232. doi: 10.3389/fpls.2021.641232 (PMC8160286; doi:10.3389/fpls.2021.641232)

Supplementary Figure 1 Map of the research area, Tatra Mountains National Park (TANAP), Slovakia (SK). Two management treatments, site without management (NEX) and site with traditional management (EXT), each with ten research plots.

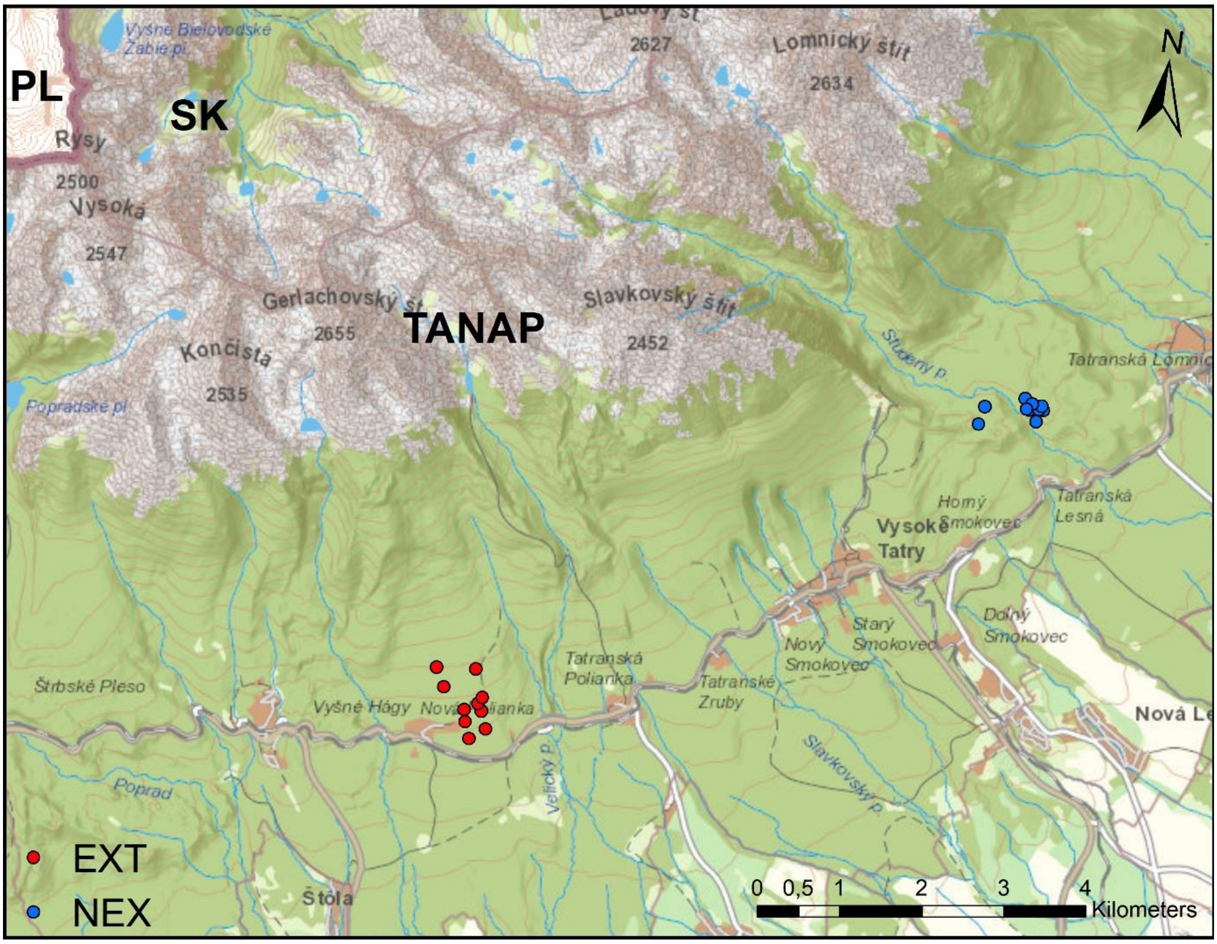

Supplement: Supplementary Figure 1 — Map of the research area, Tatra Mountains National Park (TANAP), Slovakia (SK). Two management treatments, site without management (NEX) and site with traditional management (EXT), each with ten research plots. [file Image_1.PDF]
