# Supplementary material for: Management After Windstorm Affects the Composition of Ectomycorrhizal Symbionts of Regenerating Trees but Not Their Mycorrhizal Networks
Source: Front Plant Sci. 2021 May 14;12:641232. doi: 10.3389/fpls.2021.641232 (PMC8160286; doi:10.3389/fpls.2021.641232)

Supplementary Figure 2 Rarefaction curves showing the ECM species richness on tree species (P – *Picea abies*, L – *Larix decidua*, and B – *Betula pendula*) in NEX (plots without management) and EXT (plots with traditional management) treatments.

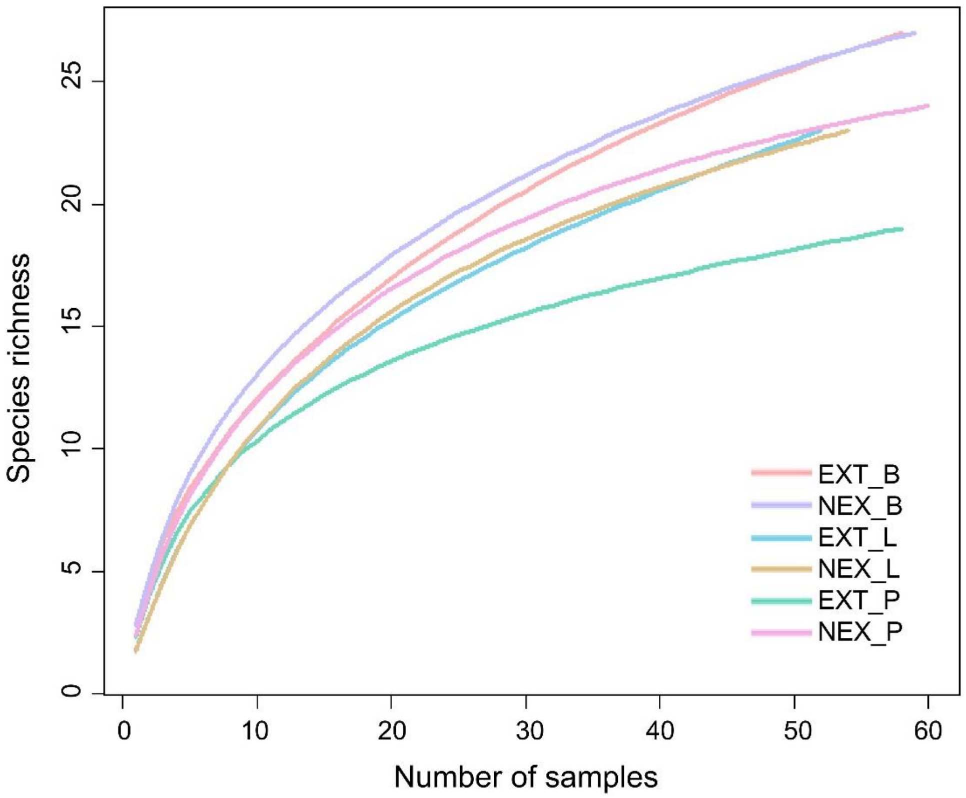

Supplement: Supplementary Figure 2 — Rarefaction curves showing the ECM species richness on tree species (P – Picea abies, L – Larix decidua, and B – Betula pendula) in NEX (plots without management) and EXT (plots with traditional management) treatments. [file Image_2.PDF]
